# Sudden Severe Bradycardia Induced by Propofol-Succinylcholine in a Healthy Adult Patient: A Case Report

**DOI:** 10.7759/cureus.69207

**Published:** 2024-09-11

**Authors:** Kinjal M Solanki, Tyler Lipscomb, Efrain Riveros Perez, Lyndon Lennard

**Affiliations:** 1 Anesthesiology, Augusta University Medical College of Georgia, Augusta, USA

**Keywords:** anesthesia induction, anticholinergic premedication, bradycardia, cardiac adverse events, propofol, succinylcholine

## Abstract

This case report describes a 27-year-old healthy female patient undergoing bilateral salpingectomy for sterilization who experienced sudden bradycardia following induction with propofol (2, 6 diisopropylphenol) and succinylcholine. Despite lacking prior cardiac issues, the patient's heart rate dropped precipitously, necessitating intervention with glycopyrrolate. This case report highlights the importance of premedication with anticholinergics to prevent bradycardia in similar scenarios, underscoring the need for vigilance and preparedness to manage adverse cardiac events during anesthesia induction, even in patients without evident risk factors.

## Introduction

Propofol and succinylcholine are widely used anesthetic agents worldwide. Propofol (2, 6 diisopropylphenol) is the most popular induction agent with commendatory characteristics of rapid and smooth induction and recovery with minimal side effects. Propofol decreases blood pressure, cardiac output, and systemic vascular resistance [1], due to the inhibition of sympathetic vasoconstriction and impairment of the baroreceptor reflex regulatory system [1,2].

Currently, succinylcholine remains the only depolarizing muscle relaxant in clinical use. Its continued popularity, despite known complications, is due to its rapid onset (deep block within 60 seconds), short duration of action, production of non-toxic metabolites, and low cost [3]. However, succinylcholine mimics acetylcholine, stimulating muscarinic and nicotinic receptors, which can lead to serious side effects including bradycardia, asystole, muscle fasciculations, increased intragastric, intraocular, and intracranial pressures, prolonged paralysis in patients with plasma pseudo choline esterase deficiency, and malignant hyperthermia [4]. Nonetheless, its brief duration of action makes it a preferred muscle relaxant in scenarios of anticipated difficult intubation. Although both agents can cause bradycardia, this adverse event is not universal and can be prevented with premedication. We are presenting a case of sudden bradycardia in a young, healthy patient without any history of previous cardiac disease.

## Case presentation

A 27-year-old female with G10P4A0S6L4 (gravida 10, para 4, abortion 0, stillbirth 6, live birth 4) was posted for bilateral salpingectomy for sterilization. The patient was recently admitted to the labor and delivery department for labor and spontaneous rupture of membranes on March 8th, 2024, at 38.4 weeks of gestation. Her medical history includes asthma for which she does not need a daily inhaler, post-traumatic stress disorder, anxiety, and depression, for which the patient was prescribed Tab. fluoxetine 60 mg, and iron deficiency anemia. She has a history of a C-section in her gravida 3 due to a breech position. She does not have any known drug allergies.

On examination, her BMI was 33.9 kg/cm2. Preoperative examination includes normal temperature with HR of 60 beats/ min and BP of 106/74 mmHg. On airway examination, the patient was edentulous with normal temporomandibular joint mobility, patent nares, and normal mouth anatomy. The patient was MPG 2 (Mallampati grade) with a full range of neck motion. On systemic examinations, the respiratory, cardiovascular, and central nervous systems appeared normal. Her complete blood count and EKG were within normal limits. In the operation theatre, the heart rate was 88 beats/ min, and BP was 140/90 mmHg. All American Society of Anesthesiologists (ASA)-standardized monitors were attached, and IV fluids were started.

**Figure 1:**
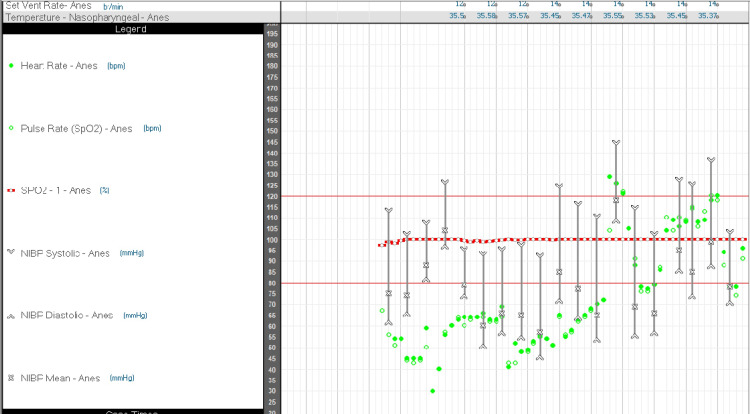
Intraoperative hemodynamic parameters The figure depicts heart rate (HR), systolic (SBP) and diastolic blood pressure (DBP), mean arterial pressure (MAP), and oxygen saturation (SpO2) during the intraoperative period. The figure shows a drop in the heart rate <30 beats/ min at one point.

Following pre-oxygenation for three minutes, she was induced with Inj. fentanyl 175 mcg, Inj. lignocaine 1% 7 ml, Inj. propofol 150 mg followed by Inj. succinylcholine 100 mg. Suddenly, the patient’s heart rate dropped to 41 beats/ min and 29 beats/ min (Figure 1). Immediately, Inj. glycopyrrolate 0.6 mg intravenous was administered, and the heart rate was back to 79 beats/ min. After that, the patient was intubated with a 7 mm portex endotracheal tube by direct laryngoscope with a 3 mm blade. After confirming bilateral air entry, the ET tube was fixed at the 20 cm marking, and the patient was put on pressure-controlled ventilation with a volume guarantee (PCV-VG) mode of ventilation with a tidal volume (TV) of 450 ml, respiratory rate (RR) of 12/ min, inspiration to expiration ratio (I:E) of 1:2, O2 of 60%, and flow of 1 L/min with sevoflurane as an inhalational agent. Inj. rocuronium 40mg as a muscle relaxant and Inj. phenylephrine 300 mcg was given to induce a drop in blood pressure. The surgery proceeded uneventfully; in the end, the block was reversed with Inj. sugammadex 200 mg. The patient was then extubated and experienced a typical recovery from anesthesia. At the end of the surgery, the patient was transferred to the post-anesthesia care unit, where her postoperative vitals were stable, including blood pressure of 116/72 mmHg, heart rate of 68 beats/ min, respiratory rate of 14 breaths/ min, and pulse oximetry reading of 99% on 2 liters per minute of oxygen via nasal cannula.

## Discussion

Baraka suggested that the use of a propofol-suxamethonium sequence can result in significant bradycardia in patients who have not been premedicated with atropine. Premedicating with atropine can prevent bradycardia. Unlike thiopentone, propofol lacks central vagolytic activity and may instead have a central vagotonic effect, which could enhance the muscarinic effects of suxamethonium [5].

Cullen and Sorensen's studies emphasized the importance of ensuring adequate anticholinergic premedication when using a sequence of propofol followed by suxamethonium for anesthesia induction. This precaution is particularly crucial when the induction process is supplemented with centrally vagotonic opioids such as fentanyl and its derivatives, as well as after repeated administration of suxamethonium [6,7].

Williams et al. concluded that cardiac changes induced by succinylcholine in humans are mediated via both sympathetic and parasympathetic efferent nerves [6,8]. Galindo and Davis further explained that these changes result from sympathetic post-ganglionic stimulation, which produces reflex cardiac effects via the baroreceptors and vagus nerve [9]. This finding aligns with Beretervide's observations in dogs and rabbits, where anesthesia with pentobarbitone abolished the response [10]. In young children, cardiac output is more dependent on heart rate than in adults due to their limited ability to increase myocardial contractility. This makes them more susceptible to bradycardia during anesthesia, particularly in younger children with poorer ASA physical status [11]. Keenan et al. study showed that propofol significantly reduces heart rate in children under two years old, with a bradycardia incidence of 1.27% during the first year of life, a higher rate than older, healthy individuals [11]. Despite this complication being associated with most anesthetic agents, there are rare chances of bradycardia with thiopentone, but arrhythmia may occur [1]. Thiopentone’s rapid redistribution within the body likely explains why the induction dose provides only brief protection [1]; this is why we did not use thiopentone while inducing this patient and instead opted for smooth induction with propofol. 

There are chances of blood pressure and heart rate changes after propofol in elderly people with many comorbidities, including cardiac issues. Succinylcholine-induced vital changes are common in patients with hyperkalemia and people with pseudocholinesterase enzyme deficiency. However, in our case, the patient is a healthy young adult without any comorbidities who underwent acute severe bradycardia.

## Conclusions

In this case, we observed severe bradycardia following induction with propofol and succinylcholine in a healthy adult patient without prior cardiac issues. The incident underscores the importance of considering premedication with anticholinergics like glycopyrrolate or atropine to mitigate the risk of bradycardia, particularly when using a propofol-succinylcholine sequence. This case highlights the necessity for vigilance and preparedness to manage potential adverse cardiac events, even in patients without evident risk factors.
